# Associations between susceptibility to online health misinformation, health risk behaviors and vaccine hesitancy among nursing students in Greece

**DOI:** 10.3389/fpsyg.2026.1935732

**Published:** 2026-09-21

**Authors:** Aglaia Katsiroumpa, Antigoni Kolisiati, Ioannis Moisoglou, Maria Nystazaki, Petros Galanis

**Affiliations:** 1 Clinical Epidemiology Laboratory, Faculty of Nursing, National and Kapodistrian University of Athens, Athens, Greece; 2 Department of Nursing, University of Thessaly, Larissa, Greece; 3 Department of Nursing Education “Attikon” University General Hospital, Athens, Greece

**Keywords:** fake news, health behaviors, health misinformation, nursing, students, susceptibility, vaccine hesitancy

## Abstract

**Introduction:**

The rapid spread of online health misinformation has emerged as a significant public health concern, particularly among young adults who rely heavily on digital sources for health information. Understanding how susceptibility to online health misinformation is associated with health risk behaviors and vaccine hesitancy is especially critical among nursing students, who will play a key role as future healthcare professionals. The aim of this study was to examine whether susceptibility to online health misinformation was associated with health behaviors and vaccine hesitancy among nursing students.

**Methods:**

A cross-sectional survey was conducted in Greece during September 2025 and 414 nursing students were included in the study. Susceptibility to online health misinformation was measured with the Health-Related Online Misinformation Susceptibility Scale. Health behaviors were assessed using the Health Behavior Inventory – Short Form and vaccine hesitancy was measured with the Vaccine Hesitancy Scale. Multivariable linear regression models were applied to determine the independent association between susceptibility to online health misinformation and health behaviors/vaccine hesitancy, following adjustment for several sociodemographic and behavioral confounders.

**Results:**

Multivariable analysis showed that higher levels of susceptibility to online health misinformation were significantly associated with worse diet behaviors (*p* < 0.001), worse anger and stress management (*p* < 0.001), and higher levels of substance use (*p* < 0.001). Also, higher susceptibility to online health misinformation was independently associated with greater vaccine hesitancy, reflected in lower confidence in vaccines and higher perceived vaccination risk (*p* < 0.001).

**Conclusion:**

These results suggest that susceptibility to online health misinformation is associated with less healthy behaviors and greater vaccine hesitancy among nursing students.

## 1 Introduction

The substantial global increase in access to the internet and smartphones, together with the rapid development of social media platforms, has enabled individuals to obtain immediate access to information related to their work, news, and entertainment. It is estimated that the time people spend in front of screens, regardless of the device used, exceeds 6 h per day (Singh and Mahur, 2026). Among young people, for whom social media use is particularly widespread, the time spent on these platforms is estimated to reach approximately 9 h per day (Woodward et al., 2025). Health-related topics are among the types of information most frequently sought by users online. The majority of individuals who search for health information online typically use social media platforms, do so on a weekly basis, and report that such information influences their health-related decision-making (Jia et al., 2021). The main topics searched include diet and nutrition, exercise and fitness, specific diseases, medication or treatment options, mental health issues, health promotion, and sexual and reproductive health information (Jia et al., 2021; Zhao et al., 2022).

Health information disseminated online often lacks credibility, increasing the likelihood that individuals will fall victim to misinformation, which can be defined as a health-related factual claim that is currently false due to a lack of scientific evidence (Chou et al., 2018). Misinformation should be distinguished from disinformation, which refers to the deliberate dissemination of false or misleading information for purposes such as financial gain, power, harm or reputation (Swire-Thompson and Lazer, 2020; Suarez-Lledo and Alvarez-Galvez, 2021). Research has shown that health misinformation spreads rapidly online, with fake news being shared on social media platforms, as a result of the ease with which non-experts can rapidly circulate information, together with the influence exerted by bots and algorithmic processes on social media platforms (Denniss and Lindberg, 2025). The high prevalence of misinformation is particularly evident on social networking platforms, where the proportion of health-related misinformation has been reported to range from 0.2 to 28.8% (Borges do Nascimento et al., 2022). Health misinformation posts mainly concern vaccines, drugs or smoking-related issues, noncommunicable diseases, pandemics, eating disorders, and medical treatments (Suarez-Lledo and Alvarez-Galvez, 2021).

The search for online health information often shapes users’ health-related decisions, thereby influencing both their individual health status and public health more broadly. Health misinformation negatively affects health-related behaviors by shaping inaccurate perceptions of risks, reducing trust in healthcare professionals, and increasing the acceptance of practices that are not supported by scientific evidence. Specifically, misinformation may affect citizens’ mental health, their use of health services, their dietary habits, as well as their vaccination behavior. Individuals exposed to fake news may experience a range of emotional and psychological disturbances, including confusion, fear, anger, fatigue, panic, anxiety, and depression (Rocha et al., 2023). More severe consequences have also been reported, such as increased suicidal thoughts and psychological distress (Kisa and Kisa, 2024). In addition to the consumption of misinformation, its dissemination may exert an even stronger adverse effect on mental health. In particular, users who shared COVID-19 misinformation showed an approximately twofold greater increase in anxiety compared with comparable users who did not engage in misinformation sharing (Verma et al., 2022). Socio-demographic analysis of the study Verma et al., among Twitter users, revealed that women, racial minorities, and individuals with lower levels of education in the United States experienced a disproportionately higher increase in anxiety when compared to the other users (Verma et al., 2022). Additionally, misinformation may also influence citizens’ intention to vaccinate. Despite the well-established role of vaccination as one of the most effective interventions for protecting public health, concerns regarding vaccine safety remain common among the general population. Such concerns may lead individuals to delay or avoid vaccination for themselves or their children. In this context, exposure to vaccine-related misinformation can further amplify vaccine hesitancy and contribute to vaccine refusal (Loomba et al., 2021). Although the proportion of individuals refusing vaccination as a result of misinformation may seem relatively low (Loomba et al., 2021), even this level of vaccine refusal can be sufficient to undermine herd immunity and pose a threat to public health. A recent systematic review of 36 studies synthesized how different communication channels relate to vaccine attitudes, intentions, and hesitancy. Exposure to traditional media, such as television, radio, and newspapers, was generally associated with greater vaccine adherence, whereas social media users displayed lower trust, and these associations were consistently moderated by sociodemographic characteristics including age, educational level, and political orientation (Leonforte et al., 2026a). Notably, that review excluded studies conducted specifically in healthcare populations and relied predominantly on cross-sectional evidence, underlining both the need for data on health professions students and the value of examining individual susceptibility to misinformation rather than exposure to a particular channel alone. In addition, individuals exposed to misinformation may be less likely to comply with recommendations issued by competent public health authorities, may demonstrate reduced trust in scientific committees, and may fail to adhere to disease prevention measures aimed at protecting public health, as was observed during the COVID-19 pandemic (Kisa and Kisa, 2024). Health behaviors associated with misinformation on social media that are dangerous to health and even life-threatening have also been documented, including the ingestion of disinfectants as a purported treatment for COVID-19. The use of disinfectants was even recommended by some healthcare professionals, who had themselves evidently fallen victim to misinformation (Soto Vega et al., 2022; Sauvayre, 2023). Nutrition-related misinformation represents an area in which a substantial amount of inaccurate or misleading information circulates. Consumers exposed to such misinformation often experience difficulties in recognizing it, while their dietary behaviors may also be influenced (Ruani and Reiss, 2023; Melios et al., 2025). Moreover, misinformation may also shape attitudes toward substance use, leading individuals to increased acceptance of nonmedical prescription drug use (Seo et al., 2025).

These associations can be interpreted within established health behavior theories. The Health Belief Model posits that health-related action depends on perceived susceptibility to and perceived severity of a threat, weighed against the perceived benefits of and barriers to protective behavior (Rosenstock, 1974). Online health misinformation operates directly on these appraisals: claims that trivialize the consequences of poor diet or promote unverified remedies lower perceived susceptibility and severity, while inflating the perceived benefits of ineffective alternatives, thereby weakening the motivation to adopt healthier behaviors. The same mechanism applies to vaccination, since misinformation amplifies perceived barriers and perceived risks and diminishes perceived benefits, constructs that are consistently associated with vaccine hesitancy (Limbu et al., 2022). The Theory of Planned Behavior further specifies that behavior follows from intention, which is shaped by attitudes, subjective norms, and perceived behavioral control (Ajzen, 1991); misinformation-dense social media environments distort attitudes toward unhealthy behaviors and convey the impression that such behaviors are widely endorsed by peers. Moreover, false beliefs continue to influence reasoning even after they have been corrected (Ecker et al., 2022), suggesting that highly susceptible individuals may persistently underestimate the risks of unhealthy behaviors.

Research has identified a considerable number of factors that may contribute to users’ misinformation susceptibility. With regard to socio-demographic and clinical characteristics, individuals with lower educational attainment, those living alone, those reporting symptoms of depression, and those obtaining information exclusively from non-traditional sources appear to be more likely to believe misinformation (Delmastro and Paciello, 2022). Furthermore, anxiety, pre-existing misinformation-related beliefs, repeated exposure to health misinformation, and low income have been linked to greater susceptibility to misinformation (Pan et al., 2021). In this context, mental disorders, including anxiety and depression, appear to act both as predictors of susceptibility to misinformation and as negative outcomes of misinformation exposure. This bidirectional relationship may place individuals with mental disorders at risk of becoming trapped in a vicious cycle, whereby their mental health status heightens their susceptibility to misinformation, which subsequently contributes to further deterioration of their psychological well-being. Moreover, repeated exposure to health misinformation may trigger the “illusory truth effect” (van der Linden, 2022). This process is especially pertinent to social media environments, in which inaccurate or deceptive health information can be swiftly disseminated, reinforced by numerous users, and reproduced across multiple platforms. Recurrent exposure to the same misleading assertion in different digital contexts may therefore increase its familiarity and perceived credibility, making users more inclined to regard it as valid, even when they have relevant prior knowledge or apply analytical thinking (van der Linden, 2022).

University students engage daily with multiple digital platforms and often rely on them as primary sources of health-related information (Abdoh, 2022; Oni et al., 2026). Although approximately half of the participants agreed that misleading content is common on these platforms, the remaining students perceived social media platforms as trusted sources of health information (Oni et al., 2026). When students seek health-related information online, they encounter two major challenges: the first concerns their ability to assess the reliability of such information, while the second relates to their capacity to determine whether the information has been produced or presented with underlying commercial interests (Dadaczynski et al., 2021). As in the general population, misinformation among university students has been identified as a predictor of vaccine hesitancy and reduced behavioral intention to get vaccinated, while it may also adversely affect their mental health (Lee et al., 2022; Jiang and Wang, 2024).

The majority of studies have examined the relationship between misinformation and mental health, as well as vaccination behavior. By contrast, studies focusing on dietary habits, health service utilization, and substance use remain extremely scarce, thereby highlighting a gap in the literature to which the present study may contribute empirical evidence. We focused on nursing students because, unlike students in non-health disciplines, they occupy a dual position: they are heavy social media users like their peers, but they will soon advise patients on vaccination and lifestyle, so their beliefs carry consequences beyond their own health. Importantly, clinical education oriented toward evidence-based practice does not automatically protect them from the infodemic. Fewer than half of nursing students across seven European countries intended to accept COVID-19 vaccination (Patelarou et al., 2021), vaccine hesitancy is well documented among healthcare students internationally (Mustapha et al., 2021), and healthcare professionals themselves have endorsed misinformed practices (Sauvayre, 2023). Examining susceptibility to misinformation in this group is therefore *a priori*ty rather than a redundant question. The Greek context reinforces this concern. Evidence-based practice has been reported to be formally integrated into the curricula of only a minority of Greek universities, and a recent study of Greek undergraduate nursing students found only moderate evidence-based practice competence, which did not improve with year of study (Miliara et al., 2024). Critical appraisal skills, the principal defense against online health misinformation, therefore cannot be taken for granted in this population. Moreover, to our knowledge, no study has examined susceptibility to online health misinformation among nursing students in Greece, or linked it to their own health behaviors. The present study addresses this gap. In this context, we examined the association between online health misinformation susceptibility, health behaviors and vaccine hesitancy among nursing students in Greece.

## 2 Materials and methods

### 2.1 Study design

A cross-sectional online survey was conducted in Greece in September 2025. Data collection took place at the beginning of the Greek academic year. Given the age distribution of the sample, most participants were therefore in their first or second year of study, and first-year students had recently enrolled with minimal or no exposure to nursing coursework. The questionnaire was created using Google Forms and distributed through social media platforms, including Facebook and Instagram, resulting in a convenience sample. The inclusion criteria were: (1) being a nursing student; (2) spending at least 30 min per day on the internet or social media, to ensure a minimum level of exposure to online information; and (3) providing informed consent. This study followed the Strengthening the Reporting of Observational Studies in Epidemiology (STROBE) guidelines (von Elm et al., 2007). We calculated sample size using G*Power v.3. 1.9.2. We included six covariates and one predictor in the multivariable model, an assumed effect size for the predictor–outcome association of 0.04, a statistical power of 95%, and a 5% type I error. Therefore, the estimated required sample size was 327 participants.

### 2.2 Measurements

Data on the following demographic covariates was obtained: gender (categorized as male or female), age (continuous variable), financial status (self-rated on a scale of financial well-being from 0 = very poor to 10 = excellent), digital literacy (single-item self-assessment on a scale ranging from 0 = very poor to 10 = very good), and the hours spent per day on the internet or/and social media (continuous variable).

Online health misinformation susceptibility in our sample was assessed using the Health-Related Online Misinformation Susceptibility Scale (HR-OMISS) (Katsiroumpa et al., 2026a). The HR-OMISS was adapted based on the Online Misinformation Susceptibility Scale (OMISS) (Katsiroumpa et al., 2025); OMISS measures online misinformation susceptibility in general while HR-OMISS measures specifically online health misinformation susceptibility. The HR-OMISS comprises nine items, with responses recorded on a five-point Likert scale: never (5), rarely (4), sometimes (3), very often (2) and always (1). For example, the following are three items from the HR-OMISS; “How often do you check the website domain and URL?”, “How often do you check the publication date of the post” and “How often do you check if the post includes the author’s name?”. Thus, an answer of “never” in HR-OMISS indicated the greatest level of online health misinformation susceptibility. On the other hand, an answer of “always” indicates the lowest level of online health misinformation susceptibility. Thus, the total score ranges from 9 to 45, with higher scores indicating greater susceptibility. It should be noted that the HR-OMISS is a self-report instrument that assesses digital verification behavior. Its items capture how frequently respondents apply procedural checks, such as examining the website domain, the publication date, or the presence of a named author, when they encounter health content online. The scale therefore does not measure cognitive ability, health literacy, or medical knowledge, nor does it objectively test the capacity to discriminate true from false health claims through recognition tasks. Lower reported engagement in verification behaviors is treated as an indicator of greater susceptibility to online health misinformation, consistent with evidence that source- and date-checking strategies improve discernment between accurate and false content (Guess et al., 2020). In our study, we employed the valid Greek version of the HR-OMISS (Katsiroumpa et al., 2026a), and the Cronbach’s alpha for the HR-OMISS was 0.903.

The Health Behavior Inventory – Short Form (HBI-SF) (Levant et al., 2020) was used to assess health behaviors in our sample. The HBI-SF comprises 12 items, and answers are provided on a 7-point Likert scale from 1 (strongly disagree) to 7 (strongly agree). Six items are reverse-coded so that higher scores across all items reflect greater levels of health risk. The questionnaire consists of four subscales: diet (three items), proper use of health care resources (three items), anger and stress (three items) and substance use (three items). Each subscale score is calculated as the mean of the corresponding item scores. Scores range from 1 to 7, with higher values indicating a greater risk of unhealthy behaviors. The Greek version of the HBI-SF, which has been previously validated for the Greek context was used (Katsiroumpa et al., 2026b). The Cronbach’s alpha value for the four subscales of the HBI-SF ranged from 0.713 to 0.832 in our study.

Vaccine hesitancy among our study participants was measured using the Vaccine Hesitancy Scale (VHS) (Shapiro et al., 2018). The VHS consists of 10 items, with responses provided on a 5-point Likert scale ranging from 1 (strongly disagree) to 5 (strongly agree). Seven out of 10 items are reverse coded, so that higher scores indicate greater vaccine hesitancy. The VHS consists of two subscales: lack of confidence (seven items) and risk perception (three items). Subscale scores are calculated by summing the item responses and dividing the total by the number of items, with subscale scores ranging from 1 to 5. The valid Greek version of the VHS was used (Gialama et al., 2024). In our analyses, the Cronbach’s alpha for “lack of confidence” and “risk perception” subscales was 0.911 and 0.754, respectively.

### 2.3 Ethical issues

The study protocol was formally approved by the Ethics Committee of the Faculty of Nursing at the National and Kapodistrian University of Athens (approval No. 75, July 13, 2025). The study adhered to the ethical standards included in the Declaration of Helsinki (World Medical Association, 2013). Answers were collected anonymously and on a voluntary basis, following a detailed explanation of the study’s objectives and procedures, and the acquisition of the informed consent by the participants.

### 2.4 Statistical analysis

Categorical variables are reported as frequencies and percentages. Also, we presented continuous variables using means, standard deviations (SD), medians and interquartile ranges (IQR). Normality was assessed by the Kolmogorov–Smirnov test along with Q-Q plots, skewness values, and kurtosis values, confirming normal distribution for continuous variables. The only exception was the “substance use” subscale (HBI-SF) since it shows a highly-skewed distribution, characterized by a massive “floor effect.” Our predictive/independent variable was susceptibility to online health misinformation and the predicted/dependent ones were health behaviors and vaccine hesitancy. Linear regression analysis was used due to the normally distributed dependent variables. We initially performed simple linear regression analyses to investigate univariable associations and subsequently used multivariable regression models to assess the independent effect of susceptibility on online health misinformation. Results are reported as unadjusted and adjusted beta coefficients with 95% confidence intervals (CI) and *p*-values. We measured the Variance Inflation Factor (VIF) for the independent variables to test for collinearity in the multivariable linear regression models, with a VIF higher than 5 indicating a serious collinearity issue. Variance Inflation Factors in our multivariable models were acceptable (see Tables 1, 2). Additionally, since the “substance use” subscale (HBI-SF) did not follow the normal distribution, we performed logistic regression analysis after dichotomizing the variable. In particular, we used the median value (i.e., one) of the “substance use” subscale to dichotomize our participants. Thus, participants with score from two to seven considered to be the high risk group, while participants with score equal to one considered being the low risk group. First, we performed simple logistic regression analyses, and then we constructed a final multivariable logistic regression model to identify the independent effect of susceptibility on online health misinformation. Odds ratios, 95% CIs, and *p*-values are presented in case of logistic regression analysis. Additionally, to examine whether the associations differed by gender, we added a gender × misinformation susceptibility interaction term to each multivariable model, with susceptibility mean-centered, and we additionally conducted gender-stratified analyses. First, to avoid multicollinearity issues, we calculated the centered HR-OMISS score by subtracting participants’ score from mean score (i.e., 28.64). Then, we calculated the interaction term gender × HR-OMISS score, and we included it in the final multivariable terms. Statistically significant interaction terms indicated a moderating effect of gender. Moreover, we performed gender-stratified analyses by presenting finding from the final multivariable models separately in males and females. Pearson’s correlation coefficients were used to assess correlations between normally distributed scales scores. Probability values less than 0.05 was used as the criterion for statistical significance. The IBM SPSS 28.0 (IBM Corp. Released 2021. IBM SPSS Statistics for Windows, Version 28.0. Armonk, NY: IBM Corp) was used to perform the analysis.

**Table 1:** Linear regression models with score on the Health-Related Online Misinformation Susceptibility Scale (HR-OMISS) as the independent variable and score on the Health Behavior Inventory – Short Form as the dependent variable (*n* = 414).

Dependent variables	Univariate models			Multivariable model^a^		
	Unadjusted coefficient beta	95% CI for beta	*P*-value	Adjusted coefficient beta	95% CI for beta	*P*-value
Diet^b^	0.060	0.042 to 0.078	<0.001	0.058	0.038 to 0.077	<0.001
Gender × HR-OMISS score				0.021	−0.023 to 0.066	0.340
Males				0.020	−0.028 to 0.067	0.407
Females				0.063	0.042 to 0.085	<0.001
Proper use of health care resources^c^	0.019	−0.003 to 0.042	0.094	0.013	−0.011 to 0.036	0.286
Gender × HR-OMISS score				0.029	−0.023 to 0.082	0.273
Males				0.006	−0.037 to 0.049	0.789
Females				0.025	−0.002 to 0.052	0.072
Anger and stress^d^	0.074	0.055 to 0.092	<0.001	0.078	0.058 to 0.098	<0.001
Gender × HR-OMISS score				−0.0005	−0.045 to 0.044	0.983
Males				0.093	0.058 to 0.129	<0.001
Females				0.079	0.056 to 0.102	<0.001

^a^ Models are adjusted for gender, age, financial status, digital literacy, and daily time in web/social media. ^b^ *R*^2^ for the final multivariable model = 10.1%; *p*-value for ANOVA < 0.001; Variance Inflation Factor = 1.163. ^c^ *R*^2^ for the final multivariable model = 11.0%; *p*-value for ANOVA < 0.001; Variance Inflation Factor = 1.163. ^d^ *R*^2^ for the final multivariable model = 15.7%; *p*-value for ANOVA < 0.001; Variance Inflation Factor = 1.163. CI: confidence interval.

**Table 2:** Linear regression models with score on the Health-Related Online Misinformation Susceptibility Scale (HR-OMISS) as the independent variable and score on the Vaccine Hesitancy Scale as the dependent variable (*n* = 414).

Dependent variables	Univariate models			Multivariable model^a^		
	Unadjusted coefficient beta	95% CI for beta	*P*-value	Adjusted coefficient beta	95% CI for beta	*P*-value
Lack of confidence^b^	0.025	0.016 to 0.035	<0.001	0.023	0.013 to 0.032	<0.001
Gender × HR-OMISS score				−0.001	−0.023 to 0.022	0.960
Males				0.023	−0.001 to 0.046	0.058
Females				0.025	0.013 to 0.036	<0.001
Risk perception^c^	0.023	0.013 to 0.033	<0.001	0.024	0.013 to 0.034	<0.001
Gender × HR-OMISS score				−0.010	−0.034 to 0.015	0.448
Males				0.022	−0.009 to 0.053	0.170
Females				0.021	0.009 to 0.033	0.001

^a^ Models are adjusted for gender, age, financial status, digital literacy, and daily time in web/social media. ^b^ *R*^2^ for the final multivariable model = 14.1%; *p*-value for ANOVA < 0.001; Variance Inflation Factor = 1.163. ^c^ *R*^2^ for the final multivariable model = 5.2%; *p*-value for ANOVA < 0.001; Variance Inflation Factor = 1.163. CI: confidence interval.

## 3 Results

### 3.1 Demographic characteristics

A total of 414 participants were included in the study, and most were women (82.1%). Participants’ mean age was 19.18 years (SD = 2.31), with a median value of 18 years and IQR was one. The average financial status score was 5.62 (SD = 1.70), with a median score of 6 (IQR = 2), a minimum value of 0, and a maximum value of 9. Digital literacy was moderate with a mean score of 7.67 (SD = 1.59), a median of 8 (IQR = 2), a minimum value of 2, and a maximum value of 10. Participants spent, on average, 4.29 h (SD = 2.37) per day using the internet and social media (median value = 4; IQR = 2). Demographic characteristics are detailed in Table 3.

**Table 3:** Demographic characteristics of the study sample (*n* = 414).

Characteristics	*N*	%
Gender		
Males	74	17.9
Females	340	82.1
Age^a^	19.18	2.31
Financial status^a^	5.62	1.70
Digital literacy^a^	7.67	1.59
Daily time in web/social media (hours)^a^	4.29	2.37

^a^ Mean, standard deviation.

### 3.2 Study scales

The average total score of the HR-OMISS scale was 28.64 (SD = 7.21) on a possible range of 9 to 45, that is, 54.6% of the scale range and slightly above the scale midpoint of 27. This corresponds to a mean item score of 3.18 on the five-point response metric, indicating that, on average, participants reported performing verification checks such as inspecting the website domain, the publication date, or the author’s name only “sometimes” when encountering health content online. Regarding health-related behaviors, participants reported a mean score of 4.12 regarding “diet,” 3.31 for “appropriate utilization of healthcare resources”, 4.09 for “anger and stress management”, while “substance use” was the lowest (mean = 1.66). Regarding vaccine hesitancy, the mean score for lack of confidence was 4.02 and 2.70 for risk perception. Descriptive statistics for all study scales are presented in Table 4.

**Table 4:** Descriptive statistics for the study scales (*n* = 414).

Scale	Mean	Standard deviation	Median	Interquartile range	Skewness	Kyrtosis
Health-related online misinformation susceptibility scale	28.64	7.21	30	10	−0.42	−0.04
Health behavior inventory – short form						
Diet	4.12	1.42	4.33	2	0.004	−0.53
Proper use of health care resources	3.31	1.69	3.33	3	0.20	−0.94
Anger and stress	4.09	1.48	4.33	2.33	−0.11	−0.87
Substance use	1.66	0.92	1	1.33	1.13	0.12
Vaccine hesitancy scale						
Lack of confidence	4.02	0.73	4.14	0.86	−1.27	2.26
Risk perception	2.70	0.77	2.67	1.33	0.19	−0.47

Correlation coefficients between study variables are shown in Table 5. Online health misinformation susceptibility was significantly and positively correlated with dietary behavior score (*r* = 0.307, *p* < 0.001), anger and stress score (*r* = 0.359, *p* < 0.001), substance use score (*r* = 0.274, *p* < 0.001), lack of confidence score (*r* = 0.250, *p* < 0.001) and risk perception score (*r* = 0.216, *p* < 0.001). These results indicate that those who are more susceptible to online health misinformation exhibit less favorable health-related behaviors and greater vaccine hesitancy.

**Table 5:** Pearson’s correlation coefficients for the study scales (*n* = 414).

Scale	2	3	4	5	6	7
1. Health-Related Online Misinformation Susceptibility Scale	0.307**	0.083	0.359**	0.274**	0.250**	0.216**
Health behavior inventory – short form						
2. Diet		0.271**	0.102*	0.200**	0.024	0.097**
3. Proper use of health care resources			0.071	0.189**	0.004	0.020
4. Anger and stress				0.264**	0.178**	0.261**
5. Substance use					0.049	0.001
Vaccine hesitancy scale						
6. Lack of confidence						0.087
7. Risk perception						

^*^ *p*-value < 0.05 and ^**^ *p*-value < 0.01.

### 3.3 Dependent variable: health behaviors

The results of linear regression analyses investigating the association between online health misinformation susceptibility and health-related behaviors are depicted in Table 1. Higher susceptibility to online health misinformation was significantly associated with higher scores in the diet dimension (adjusted beta = 0.058, 95% CI: 0.038–0.077, *p* < 0.001) and anger and stress management (adjusted beta = 0.078, 95% CI: 0.058–0.098, *p* < 0.001) in the multivariable linear regression models. There was also significant association for substance use in the adjusted logistic regression model (adjusted odds ratio = 1.060, 95% CI: 1.028–1.094, *p* < 0.001) confirming the results from the univariate logistic regression analysis (odds ratio = 1.064, 95% CI: 1.034–1.096, *p* < 0.001). All multivariable models were adjusted for gender, age, financial status, digital literacy and daily time spent on the web and social media. The multivariable models explained 10.1 to 15.7% of the variance in health behavior scores, i.e., 10.1% for the diet dimension, 11.0% for the proper use of health care resources, and 15.7% for the anger and stress management.

Moreover, we calculated the interaction terms between gender and the HR-OMISS, and we did not find statistically significant interactions (*p* for diet = 0.340, *p* for proper use of health care resources = 0.273, *p* for anger and stress management = 0.983, and *p* for substance use = 0.959). Gender-stratified analyses yielded associations in the same direction and of comparable magnitude in both groups (Table 1). For instance, the association between the HR-OMISS score and substance use was similar among males (adjusted odds ratio = 1.108, 95% CI: 1.010–1.217, *p* = 0.031), and females (adjusted odds ratio = 1.057, 95% CI: 1.020–1.095, *p* = 0.002). Given the small number of male participants (*n* = 74), these analyses were underpowered to detect modest interaction effects.

### 3.4 Dependent variable: vaccine hesitancy

Results of the linear regression analyses on susceptibility to online health misinformation and vaccine hesitancy are presented in Table 2. Higher levels of susceptibility were associated with higher levels of lack of confidence in vaccines (adjusted beta = 0.023, 95% CI: 0.013–0.032; *p* < 0.001) and higher perceived risk about vaccination (adjusted beta = 0.024, 95% CI: 0.013–0.034; *p* < 0.001). All models were controlled for gender, age, financial status, digital literacy and daily time spent on internet and social media. The findings suggest that greater susceptibility to online health misinformation is independently associated with higher vaccine hesitancy across both scales. The multivariable models explained 14.1 and 5.2% of the variance in lack of confidence, and risk perception, respectively.

Moreover, we calculated the interaction terms between gender and the HR-OMISS, and we did not find statistically significant interactions (*p* for lack of confidence = 0.960, *p* for risk perception = 0.448). Similarly, gender-stratified analyses yielded associations in the same direction and of comparable magnitude in both groups (Table 2). For instance, the association between the HR-OMISS score and lack of confidence was similar among males (adjusted beta = 0.023, 95% CI: −0.001–0.046, *p* = 0.058), and females (adjusted beta = 0.025, 95% CI: 0.013–0.036, *p* < 0.001). Given the small number of male participants (n = 74), these analyses were underpowered to detect modest interaction effects.

## 4 Discussion

Research on the association between online health misinformation susceptibility and health-related behaviors among nursing students remains extremely limited. To the best of our knowledge, this is the first study to examine the association between susceptibility to online health misinformation and both health behaviors and vaccine hesitancy among nursing students. The mean susceptibility score indicates that source-verification practices were applied inconsistently rather than routinely. Although this reflects a moderate rather than an extreme level of susceptibility, it is notable in a population trained to appraise evidence, and it means that roughly half of the verification opportunities available to these students were not taken.

The present study showed that nursing students exhibited moderate levels of susceptibility to misinformation, which were significantly associated with poorer dietary behaviors, poorer anger and stress management, and higher levels of substance use. In addition, greater susceptibility to online health misinformation was independently associated with higher levels of vaccine hesitancy. Our finding regarding misinformation and vaccine acceptance is consistent with those of previous studies, which have shown that exposure to misinformation and conspiracy theories is associated with vaccine hesitancy among students (Sallam et al., 2021; Lee et al., 2022), including healthcare students (McCready et al., 2023), and general population (Moisoglou et al., 2026).

University students constitute a population particularly susceptible to misinformation due to their specific characteristics, such as high levels of exposure to social media and dependence on these platforms, which represent a primary channel for the dissemination of fake news (Salari et al., 2025; Taha et al., 2025). Greater engagement across multiple social media platforms may increase students’ subsequent tendency to accept false information as accurate, because repeated exposure to the same content through diverse channels can strengthen its perceived credibility (“illusory truth effect”) (van der Linden, 2022). Moreover, students’ dependence on social media has been associated with mental health problems, which, in turn, may constitute predictors of susceptibility to misinformation (Feng et al., 2025). Taken together, the aforementioned factors establish conditions that facilitate the development of university students’ susceptibility to misinformation. Susceptibility to health misinformation may operate through interacting cognitive-affective pathways. Repetition increases processing fluency and familiarity, producing an illusory truth effect that makes inaccurate claims appear credible (Hassan and Barber, 2021; Udry and Barber, 2024). Distorted risk appraisals can reduce protective behaviors and normalize unhealthy choices. Misinformation-related distress may promote threat-focused information seeking, creating a feedback loop of repeated exposure and anxiety (Lee et al., 2020). Fear-arousing narratives heighten threat salience and intuitive processing, reinforcing acceptance and sharing (Xue et al., 2024). For vaccination, exaggerated safety threats and distrust shift risk–benefit judgments toward hesitancy or refusal (Lee et al., 2022).

A targeted question is whether healthcare students should differ from students in other disciplines. Two opposing forces are at play. On the one hand, exposure to biomedical content, research methodology, and evidence-based practice should strengthen critical appraisal and reduce susceptibility. On the other hand, our participants were predominantly in the first years of study (mean age 19.18 years), so this training had barely begun, while evidence-based practice remains unevenly embedded in Greek nursing curricula (Miliara et al., 2024). At the same time, their information environment does not differ from that of their peers. A further consideration is that a developing professional identity may increase confidence in one’s ability to evaluate health information without a corresponding increase in actual discernment; overconfidence in news judgement is itself associated with greater susceptibility to false content (Lyons et al., 2021). Consistent with this, our participants rated their digital literacy relatively highly (mean 7.67 out of 10) yet obtained a mid-range susceptibility score (28.64 out of 45). Health professions training should therefore not be assumed to confer protection, and a direct empirical comparison with non-health students remains an open question.

The timing of data collection warrants specific attention when interpreting these findings. The survey was administered in September 2025, at the start of the Greek academic year, and the age distribution of the sample (median 18 years, IQR 1) indicates that most participants had recently entered university, with first-year students having had minimal or no exposure to nursing coursework. Our findings should therefore be read as characterizing nursing students at the point of entry rather than as an assessment of students shaped by health sciences education. Three implications follow. First, the susceptibility levels and health behaviors reported here largely reflect patterns established before nursing education began, and are attributable to the wider information environment rather than to any shortcoming of the curriculum. Second, this makes the findings informative as a baseline: they describe the profile with which students arrive, and identify the entry point of the program as the appropriate moment for intervention, before habits consolidate. This is consistent with evidence that the transition into higher education is itself a period of adverse change in health behaviors (Winpenny et al., 2018). Third, our data cannot speak to whether susceptibility declines as students progress through the curriculum; longitudinal follow-up of the same cohort would be required, and would also indicate whether nursing education attenuates the associations reported here. Participants nonetheless differ from recent secondary school graduates in general, in that they have self-selected into a health professions program, a choice that plausibly carries distinct motivations and health-related orientations.

The susceptibility of nursing students to misinformation is of critical importance, given its substantial implications for public health. Although the present cohort is at the beginning of its training, these students are on a trajectory toward professional practice. As future healthcare professionals, nurses will be expected to educate citizens and patients on issues related to disease prevention and health promotion. Physicians and nurses are among the healthcare professionals most frequently consulted and trusted by citizens and patients for vaccine-related advice (Galanis et al., 2022a, 2022b). Evidence indicates that patients who rely on nurses or physicians as sources of vaccine information are more likely to be vaccinated (Dafnou et al., 2025). Consequently, nursing students who are exposed to misinformation and whose vaccination behavior is negatively affected may subsequently disseminate inaccurate vaccine-related information to patients and the public, thereby negatively influencing their vaccination behavior.

The findings of the present study showed that students’ susceptibility to misinformation was associated with their dietary behaviors, anger and stress management, and substance use. These findings are consistent with evidence reported in previous studies (Ruani and Reiss, 2023; Jiang and Wang, 2024; Rocha-Ávila et al., 2025). A recent cross-sectional study of 2,001 Italian university students provides a directly comparable picture of how nutrition-related decisions are informed. The internet and social media were the leading source of information on supplement-related risks, cited more frequently than healthcare professionals, while personal trainers were the most common source of recommendation and physicians accounted for only a small minority of cases (Leonforte et al., 2026b). Notably, self-rated knowledge was the strongest correlate of supplement use, even though objective knowledge was not assessed, and enrolment in health-related disciplines was not independently associated with use. Both observations parallel our own: our participants rated their digital literacy relatively highly yet obtained mid-range susceptibility scores, and health professions training did not appear to confer protection. Taken together, the two studies indicate that unregulated online content shapes the nutritional choices of university students irrespective of academic discipline, and that self-assessed competence is a poor proxy for the capacity to evaluate that content critically. The present findings may be understood within the broader context of the potentially detrimental association between misinformation exposure and mental health. Both the consumption and circulation of inaccurate or misleading content may compromise psychological well-being through multiple pathways, including the intensification of perceived fear. Such exposure may lead individuals to exaggerate the seriousness, likelihood, or personal significance of an apparent threat, consequently contributing to heightened fear, stress, social isolation, and psychological distress. For example, inaccurate information about an infectious disease may create the impression that individuals are at immediate risk or that the disease is associated with high mortality, even when it is not easily transmissible, has a low fatality rate, or is geographically confined to a distant region. In this way, misinformation may distort risk perception and contribute to heightened emotional responses (Lee et al., 2020; Hammad and Alqarni, 2021). During the COVID-19 pandemic, reliance on credible news sources among health sciences students was associated with greater knowledge of COVID-19 and lower perceived stress compared with students who did not rely on such sources (Richard et al., 2023). Another mechanism through which misinformation may contribute to mental health problems is the adoption of inappropriate health-related decisions, such as the use of medications, dietary supplements, or specific dietary regimens for the prevention or treatment of a disease. Individuals may ultimately realize that these practices not only fail to provide any benefit but may also be harmful to their health (Ruani and Reiss, 2023), thereby eliciting feelings of anger, fear, or stress (Lee et al., 2020).

With regard to substance use, scientifically unsupported information appears to be widespread among college students. Students seem to face difficulties in evaluating the credibility of the information they receive, which may contribute to the use of potentially harmful or addictive substances and products, including e-cigarettes and cannabis (Pokhrel et al., 2015; Dobbs et al., 2020; Kennedy et al., 2024). Moreover, susceptibility to misinformation has consequences that extend beyond the individual level. It may also affect public health, as the dissemination of scientifically unsupported information can influence others, particularly since young people often seek information about substance use within their peer networks (Dobbs et al., 2020). The high prevalence of misinformation makes it imperative to implement interventions aimed at promoting the acceptance and adoption of evidence-based scientific knowledge among nursing students. In particular, educational interventions have been shown to improve nursing students’ knowledge, leading to higher rates of vaccine acceptance and promoting healthy dietary habits (Torabizadeh et al., 2020; Berenson et al., 2021; Mitchell et al., 2021; Zaghamir and Ibrahim, 2023). The scale of this educational gap has recently been quantified at the European level. A mapping review of 367 digital public health training initiatives delivered across five European countries and major international organizations between 2020 and 2025 found that provision has expanded rapidly but remains fragmented and dominated by short formats: conference sessions and webinars accounted for more than three quarters of initiatives, degree programs for only 3.3%, and academic institutions for less than one third of all provision (Gorga et al., 2026). Critically for the present study, training addressing the infosphere and the spread of information over digital networks was the least represented content domain of all, appearing in just 2.5% of initiatives and remaining consistently low throughout the period examined. Health misinformation is therefore almost entirely absent from formal digital health curricula, precisely at a point when our findings indicate that susceptibility to it is associated with measurable differences in health behaviors and vaccine confidence. Recently, the Greek Minister of Education announced the pilot introduction of educational programs for lower and upper secondary school students designed to enhance their ability to recognize and critically assess fake news (Hellenic Ministry of Education, 2025). It would be important for the implementation of such programs to continue at the university level, as knowledge and skills may diminish over time.

The findings of the present study showed no significant independent association between misinformation susceptibility and the proper use of healthcare resources. The absence of an independent association with proper healthcare resource use may reflect the different determinants of service utilization. Unlike diet, stress management, or vaccination attitudes, which depend strongly on individual beliefs and risk perceptions, healthcare use is also constrained by symptom severity, service availability, cost, accessibility, and professional recommendations. An alternative explanation may be that individuals who are influenced by misinformation and perceive their health as being at risk may be more likely to seek information or “treatment” through online sources, instead of using healthcare services or consulting a healthcare professional. Moreover, nursing students may possess a certain degree of knowledge regarding the issue that concerns them or may be able to informally consult a healthcare professional, thereby avoiding the use of healthcare services.

The direction of the associations reported here cannot be established from cross-sectional data, and a reverse pathway is theoretically plausible. Rather than misinformation degrading health behaviors, pre-existing behaviors may shape what students find credible. Motivated reasoning describes how the desire to reach a particular conclusion biases the search for and evaluation of evidence, while leaving the reasoner with a subjective sense of objectivity (Kunda, 1990). A student who already eats poorly, uses substances, or has decided against vaccination faces an inconsistency between that behavior and the health knowledge to which their studies expose them, and dissonance theory predicts that such inconsistency generates pressure toward resolution (Festinger, 1962). Because changing the behavior is effortful, the cheaper resolution is to adjust the belief: content questioning nutritional guidelines, minimizing the harms of cannabis, or emphasizing vaccine adverse events supplies precisely the justification required. A comparable pattern has been described among university students who smoke, among whom dietary supplement use has been interpreted as an attempt to offset the perceived harms of smoking rather than to modify the behavior itself (Leonforte et al., 2026b). Confirmation bias then sustains the process, as information consistent with existing positions is sought preferentially and scrutinized less rigorously than information that challenges them (Nickerson, 1998), and misleading claims retain influence even after correction (Ecker et al., 2022). Two features of our data are consistent with this account. The association was strongest for anger and stress management and for diet, domains in which behavior is habitual and effortful to change, and weakest for the use of healthcare resources, which is externally constrained and offers less scope for belief-based justification. Recommendation algorithms plausibly amplify the pathway, since engagement with belief-consistent content increases subsequent exposure to similar material, so that the two directions may operate as a reinforcing cycle rather than as competing explanations. Distinguishing them requires longitudinal or experimental designs, and we accordingly frame our findings as associations rather than as evidence that misinformation causes poorer health behaviors.

The proportion of variance explained by our models warrants explicit comment. The multivariable models accounted for between 5.2 and 15.7% of the variance in the outcomes examined, meaning that most of the variability in health behaviors and vaccine hesitancy is attributable to factors we did not measure. This is expected. Health behaviors are multiply determined by genetic, familial, socioeconomic, cultural, and psychological influences, and susceptibility to online misinformation was never expected to be a dominant determinant. Our aim was to estimate the magnitude of one association, not to build a predictive model. Expressed in interpretable terms, the effects are not trivial: a one standard deviation increase in susceptibility (7.21 points) corresponds to a 0.42-point higher diet risk score and a 0.56-point higher anger and stress score on scales ranging from 1 to 7, equivalent to approximately 0.29 and 0.38 standard deviations, respectively. Across the full observed range of the susceptibility scale, the predicted difference in the anger and stress score approaches half of the scale range. Effect sizes of this order correspond to correlations that are considered meaningful in behavioral research, and expressing them as squared proportions of variance systematically understates their practical importance (Funder and Ozer, 2019). At a population level, modest shifts of this magnitude across an entire student cohort remain relevant to public health, particularly for behaviors that tend to persist into adulthood.

The magnitude of the lack of confidence score merits specific comment. A mean of 4.02 on a 1 to 5 scale indicates that participants leaned on average towards agreement with statements expressing doubt about vaccines, which is a striking finding in a cohort preparing to enter the health professions. Two contextual factors are relevant. First, this cohort’s formative exposure to vaccination coincided with the COVID-19 pandemic and its aftermath, a period during which public confidence eroded internationally, alongside falling routine immunization coverage and the resurgence of vaccine-preventable diseases (Sophian et al., 2026). Nurses are themselves an affected group rather than a protected one. A systematic review and meta-analysis focusing exclusively on nurses identified lack of confidence in vaccine safety and effectiveness as one of the most frequent determinants of hesitancy in this profession, and estimated pooled influenza vaccine uptake among nurses at only 44% (Locatelli et al., 2025). Second, our participants had only just left secondary education, where immunization is not systematically addressed as a topic, and had not yet been taught public health at university. In the absence of formal grounding, vaccine-related beliefs are likely to have been shaped predominantly through the online environment assessed in this study.

Several limitations should be acknowledged when interpreting the findings of the present study. First, the cross-sectional design precludes the establishment of causal relationships between susceptibility to online health misinformation, health-related behaviors, and vaccine hesitancy. Reverse causation is also possible, and the mechanisms through which prior behavior may shape belief are discussed above. Second, participants were recruited exclusively through Facebook and Instagram using convenience sampling, and eligibility required at least 30 min of daily internet or social media use. This sampling frame is a particular concern in a study of online misinformation, because students who use social media rarely or not at all were excluded by design and could not be represented. The sample therefore probably overrepresents students with high digital engagement and frequent exposure to online health information. Two consequences should be distinguished. First, the descriptive estimates reported here, including the mean susceptibility score, cannot be generalized to the full population of Greek nursing students. Second, because the contrast between high and low social media exposure could not be captured, this restriction of range is more likely to have attenuated than inflated the observed associations, so the reported effect sizes may be conservative. We note, nonetheless, that susceptibility to online health misinformation is a meaningful construct primarily among individuals who actually encounter such content. Future studies should recruit through university registries, institutional email lists, and classroom-based approaches, in order to include students across the full spectrum of social media use. Furthermore, the study was conducted exclusively among nursing students in Greece, while the sample consisted predominantly of females and relatively young participants, thereby limiting the generalizability of the findings to nursing students in other countries, students from other academic disciplines, and more gender- and age-diverse populations. Third, all study variables were assessed using self-reported measures administered at the same time, increasing the possibility of recall bias, social desirability bias, and common-method variance. These biases may be particularly relevant to sensitive behaviors such as substance use and to self-assessments of digital literacy, financial status, and daily internet use. Under-reporting of substance use in self-administered surveys is well documented (Latkin et al., 2017), and the distribution of substance use scores in our sample is consistent with this concern: the mean score of 1.66 on a 1 to 7 scale, with a median of 1, indicates a pronounced floor effect that restricts variance in this outcome. If under-reporting is unrelated to misinformation susceptibility, this restriction would attenuate rather than inflate the association we report, meaning that the true relationship may be stronger than estimated. Differential under-reporting cannot be excluded, however, since students who are more receptive to misinformation may also differ in their sensitivity to perceived social norms. The anonymous, self-administered online format may have mitigated, but is unlikely to have eliminated, this bias. Relatedly, susceptibility was operationalized through self-reported verification behaviors rather than through performance-based tasks in which participants classify actual true and false health claims. Self-assessed and performance-based indicators of misinformation detection do not necessarily coincide (Lyons et al., 2021), so our estimates may not fully correspond to students’ actual ability to detect health misinformation. Future studies should combine self-report scales with objective recognition tasks. The predominance of females in the study sample should also be acknowledged as a limitation. Specifically, 82.1% of participants were females, which may partly reflect the gender distribution typically observed in nursing education. However, the relatively small number of male participants limits the generalizability of the findings to male nursing students. Although we tested gender × susceptibility interaction terms and conducted gender-stratified analyses, these were underpowered given the small number of male participants, and effect modification by gender therefore remains inconclusive. Future studies should recruit gender-balanced samples designed a priori to test for such differences. Moreover, residual confounding due to unmeasured variables should also be considered. Although the multivariable models were adjusted for gender, age, financial status, digital literacy, and daily time spent on the internet and social media, several potentially important confounders were not assessed. Three variables that are central to the interpretation of vaccine hesitancy were not assessed, and their omission warrants specific attention. First, objective health literacy was not measured. Health literacy is an established determinant of vaccine attitudes, intention, and uptake (Lorini et al., 2018), and it plausibly influences both the ability to evaluate online health claims and the adoption of health behaviors; the associations reported here may therefore partly reflect underlying differences in health literacy rather than susceptibility to misinformation as such. Second, trust in scientific and public health institutions was not recorded. Trust in science is robustly associated with vaccine confidence at both the individual and the societal level (Sturgis et al., 2021), and it is likely to be associated with acceptance of online health claims as well; failure to adjust for trust may consequently have inflated the observed association between misinformation susceptibility and vaccine hesitancy. Third, previous vaccination history was not collected, although students with incomplete vaccination records or prior negative vaccination experiences may be both more hesitant and more receptive to misinformation. Because these variables were unmeasured, the reported associations should be regarded as adjusted only for the covariates listed above and not as estimates of an independent causal effect. Future studies should incorporate validated measures of health and vaccine literacy, institutional and science-related trust, and documented vaccination history, ideally with mediation analysis to determine whether misinformation susceptibility retains an independent association once these factors are accounted for. Digital literacy was assessed with a single self-rated item rather than a validated instrument or a performance-based task. Self-reported eHealth literacy correlates poorly with actual appraisal performance (van der Vaart et al., 2011), and those with the weakest skills tend to overestimate them most (Kruger and Dunning, 1999). Adjustment for this covariate is therefore incomplete, and residual confounding by actual fact-checking ability cannot be excluded. If genuine competence is inversely associated with both susceptibility and health-risk behaviors, this would tend to inflate rather than attenuate our estimates. Future studies should combine validated literacy instruments with objective performance tasks. A specific limitation concerns academic year, which was not recorded. Students in later years have greater exposure to clinical training and health sciences content and might therefore differ in both misinformation susceptibility and health behaviors. Three considerations partially mitigate, but do not remove, this concern. First, all multivariable models were adjusted for age, which is closely tied to year of study in Greek undergraduate nursing programs, where students typically enroll at 18 years; age adjustment therefore captures part of the variance attributable to academic year. Second, the sample was highly homogeneous in this respect (mean age 19.18 years, median 18 years, IQR 1), indicating that most participants were in the early years of study. This restricted range limits the extent to which academic year could confound the observed associations, while also meaning that our findings describe primarily junior students. Because data were collected in September, at the start of the academic year, first-year participants had effectively not yet been exposed to the curriculum, and our results should not be interpreted as reflecting the influence of nursing education. Third, a study of Greek undergraduate nursing students found that evidence-based practice competence did not differ significantly by academic year (Miliara et al., 2024), suggesting that curricular progression does not automatically strengthen critical appraisal skills. We nonetheless acknowledge the absence of this variable as a limitation. Future studies should record academic year and clinical placement experience, include them as covariates, and perform stratified analyses. Finally, political orientation, which was not measured in this study, may shape attitudes toward public health authorities, vaccination, and online information sources. Depending on the direction and strength of these associations, the omission of these variables may have either inflated or attenuated the observed associations. Future studies should include these factors to better isolate the independent association between online health misinformation susceptibility, health behaviors, and vaccine hesitancy. We should emphasize that the external validity of our findings is limited. The study was based on a convenience sample of nursing students in Greece, with a predominance of female participants. Therefore, the results cannot be generalized with confidence to male-dominated healthcare student cohorts, other healthcare disciplines, nursing students in other European or non-European countries, or students enrolled in non-health-related academic programs. Differences in gender composition, health sciences education, institutional trust, public health communication, vaccination policies, cultural context, and exposure to online health information may influence both susceptibility to online health misinformation and the outcomes examined in this study. Consequently, the magnitude and direction of the observed associations may differ across student populations and national contexts. Future research should employ probability-based sampling approaches, such as stratified random sampling or multi-stage sampling across universities and academic departments, to obtain more representative samples. Such approaches would allow researchers to better capture variation by gender, academic discipline, year of study, geographic region, and country, thereby improving the generalizability of findings. Finally, given the small number of male participants (*n* = 74), analyses regarding the interaction terms and stratification analyses were underpowered to detect modest interaction effects and should be interpreted with caution. Future studies with more males participants should further add significant information regarding the association between misinformation susceptibility, risk behaviors, and vaccine hesitancy separately among males and females.

## 5 Conclusion

This study provides evidence that greater susceptibility to online health misinformation is associated with less favorable health behaviors and increased vaccine hesitancy among nursing students in Greece. Specifically, higher susceptibility was independently associated with poorer dietary behaviors, less effective anger and stress management, greater substance use, lower confidence in vaccines, and higher perceived vaccination risk. Rather than generic calls for awareness, nursing curricula should adopt approaches with demonstrated efficacy. First, psychological inoculation, or prebunking, in which students learn to recognize common manipulation techniques before encountering them (Roozenbeek et al., 2022); this is well suited to delivery through serious games and role-play, and gamified formats have been shown to confer measurable resistance to online misinformation (Roozenbeek and van der Linden, 2019). Second, structured source-verification routines such as lateral reading, which correspond to the behaviors measured here and respond to brief media literacy instruction (Guess et al., 2020), combined with accuracy prompts that direct attention to veracity before engagement (Pennycook et al., 2021). Third, practical infodemiology laboratories in which students learn to formulate clinical questions and retrieve evidence from PubMed, the Cochrane Library, and official institutional portals, reducing default reliance on social media and commercial search engines; structured interventions of this kind improve evidence-based practice competencies in undergraduate nursing students (Patelarou et al., 2020). Since our participants were surveyed at university entry and showed no evident protective effect of professional training, such instruction should begin in the first year, be embedded within existing evidence-based practice teaching, use authentic misleading material on nutrition, substance use, mental health, and vaccination, and be evaluated with objective recognition tasks rather than self-rated confidence.

## Data Availability

The raw data supporting the conclusions of this article will be made available by the authors, without undue reservation.
